# Eosinophilic Esophagitis Complicated by an Esophageal Stricture in a 15-Month-Old Child

**DOI:** 10.1155/crpe/6685350

**Published:** 2025-08-05

**Authors:** Jennifer S. Hong, Samra Blanchard, Anupama Kewalramani, Elaine Leonard Puppa, William Twaddell, Nidhi Rawal

**Affiliations:** ^1^Department of Pediatrics, Division of Pediatric Gastroenterology & Nutrition, University of Maryland School of Medicine, Baltimore, Maryland, USA; ^2^Department of Pediatrics, Division of Pediatric Allergy & Immunology, University of Maryland School of Medicine, Baltimore, Maryland, USA; ^3^Department of Pathology, University of Maryland School of Medicine, Baltimore, Maryland, USA

## Abstract

Eosinophilic esophagitis (EoE) is a chronic, immune-mediated disorder characterized by dysphagia, food impactions, and esophageal eosinophilia, which can lead to fibrosis and the formation of strictures. While fibrostenotic complications are relatively rare in children, studies have shown that up to 86% of adults with EoE experience dysphagia and esophageal narrowing, compared to only 6% in children. Furthermore, the incidence of stricture formation in children remains low, with reported rates of approximately 3.4%. The youngest child previously documented with this complication was 3 years old. Risk factors for fibrostenosis in EoE include older age, prolonged disease duration, and increased symptom frequency. This represents the youngest known presentation of such a case and suggests that fibrostenosis may be a distinct phenotype of the disease, rather than merely a progression from the inflammatory form. This case underscores the importance of early and thorough evaluation of EoE, given the potential for early stricture development.

## 1. Introduction

Eosinophilic esophagitis (EoE) is a chronic, immune-mediated disorder characterized by esophageal eosinophilia, fibrosis, and stricture formation, which can lead to dysphagia and food impactions if left untreated. EoE-related strictures are most commonly observed in the proximal esophagus, with some cases also demonstrating long-segment narrowing and decreased esophageal compliance [1]. Fibrostenotic disease is thought to be age-dependent [2], suggesting that the natural progression of EoE involves inflammation leading to fibrostenosis over time. For example, studies have reported an incidence of stricture and food impaction in 25%–100% of adults presenting with dysphagia [1]. However, fibrostenotic complications are rare in children. A longitudinal adult study found that 86% of individuals with dysphagia had esophageal narrowing [3], compared to only 6% in a separate analysis involving pediatric populations [4]. Kory et al. [5] reported a 3.4% incidence of esophageal strictures in children. Traditionally, strictures are not observed until later in childhood, with the youngest reported stricture development in the setting of EoE being 3 years [6]. In this report, we describe a case of a 15-month-old with dysphagia, diagnosed with EoE and a distal esophageal stricture.

## 2. Case

A 6-month-old female with a history of prematurity at 25 weeks of gestation presented for a second opinion in the evaluation of gastroesophageal reflux (GER), cow's milk protein allergy (CMSPA) and feeding difficulties. By the time of evaluation, she had been transitioned to cow's milk-based formula given that the CMSPA diagnosis was based upon reflux presentation and symptoms persisted despite transition to an amino acid–based formula. GER was treated with ranitidine and erythromycin. Medications were continued and the diet was advanced to Stage 2 baby foods. By 10-11 months, parents noticed difficulty advancing her diet to Stage 3 baby foods and table foods. Dysphagia, refusal of higher texture foods, and vomiting developed by 13-14 months of age. A proton pump inhibitor (PPI) was started for the treatment of GER. An upper GI (UGI) series obtained at 2 months of age due to chronic GER was reviewed and did not show anatomic anomalies including stricture; however, GER was noted during the study.

**Figure 1:**
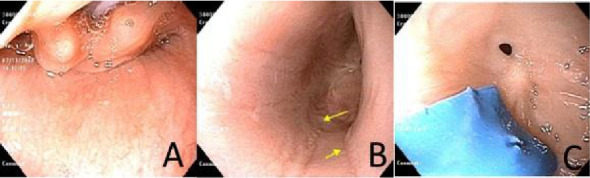
(A) Normal epiglottis at index endoscopy. (B) Longitudinal furrows in the mid-esophagus. (C) Pinhole stricture at 20 cm from incisors with a soft plastic foreign body.

However, a repeat UGI completed at age 13 months old due to her progressive symptoms of vomiting and feeding difficulties demonstrated 10–20 mm luminal narrowing of the distal esophagus, with dilation of the mid-esophagus. At 15 months, she underwent upper endoscopy while on PPI therapy (2 mg/kg/day), which revealed a significant intrinsic esophageal stricture located 20 cm from the incisors, measuring less than 10 mm in diameter. A soft, blue plastic foreign body was incidentally found and removed. Endoscopic findings included mild mid-esophageal dilation, extensive scarring, and longitudinal furrowing (Figure 1). The stricture was successfully dilated using a 6-7-8 mm through-the-scope (TTS) balloon.

**Figure 2:**
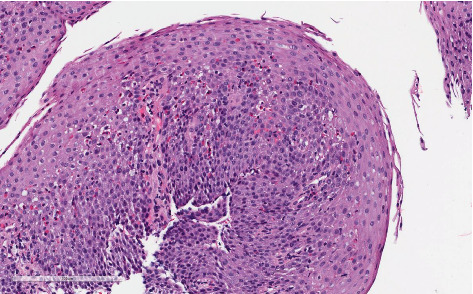
Histopathology of mid-esophagus at the index endoscopy demonstrating eosinophilic infiltrate, surface layering, and degranulation.

Esophageal biopsies obtained during upper endoscopy confirmed a diagnosis of EoE, with a peak eosinophil count exceeding 50 eosinophils per high-power field (eos/hpf; hpf = 0.096 mm^2^). Histologic features included eosinophil degranulation, surface layering, and eosinophilic microabscesses in the mid-esophagus. EoE Histologic Scoring System (HSS) assessment yielded a grade of 9/24 and a stage of 9/24 (Figure 2). Biopsies from the distal esophagus were not obtained due to a significant esophageal stricture.

In light of the severe disease phenotype characterized by a tight esophageal stricture, food impactions, dysphagia, and early age of onset, systemic corticosteroid therapy with prednisone was initiated and tapered over a 4-week period.

**Figure 3:**
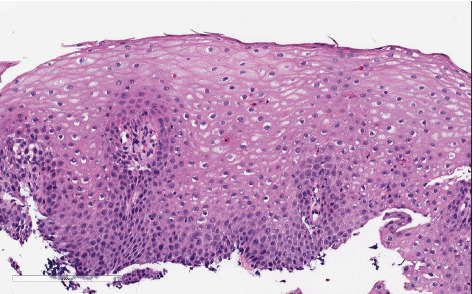
Histopathology of mid-esophagus 3 months after the index endoscopy after completing prednisone taper demonstrating improved eosinophilia.

**Figure 4:**
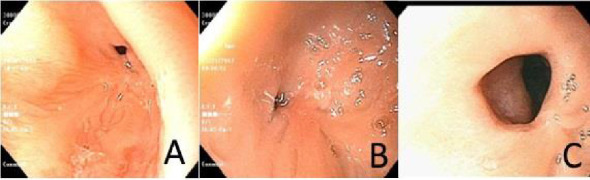
(A) 3 months after index endoscopy, after completion of prednisone taper, on PPI therapy. Stricture at 20 cm from the incisors, 8 mm in diameter. (B) 4 months after index endoscopy. Stricture 8 mm in diameter, on PPI therapy. (C) 8 months after index endoscopy. Stricture measuring 15 mm in diameter on dietary elimination therapy.

Follow-up endoscopy, performed 4 weeks after completing the prednisone course while the patient remained on PPI therapy, demonstrated histologic improvement. The eosinophil count had decreased to 8 eos/hpf in the mid-esophagus, with associated reactive epithelial changes (EoE HSS Grade 4/21, Stage 4/21) (Figure 3). However, significant luminal narrowing persisted (Figure 4). Esophageal dilation was performed using a TTS balloon (7-8–10 mm), resulting in symptomatic relief and the patient's ability to tolerate a table food diet.

Following this, long-term management was initiated. She was placed on an empiric dietary elimination of milk, egg, nuts, and peanuts based on patch testing, in conjunction with continued PPI therapy at a dose of 2 mg/kg/day. This resulted in further histologic improvement in esophageal eosinophilia to 12 eos/hpf in the distal esophagus and 5 eos/hpf in the mid-esophagus (EoE HSS Grade 5/24, Stage 4/24). Serial dilations progressively improved the esophageal stricture, which was ultimately dilated to 18 mm during the third and final procedure.

**Figure 5:**
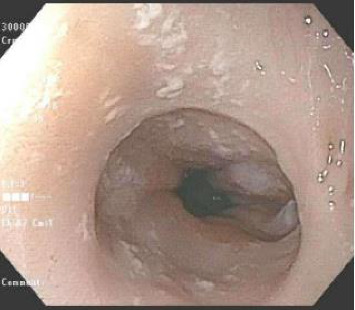
15 months after index endoscopy, while on PPI therapy and OVB. Step-off noted at the site of prior stricture. White specks seen in the mucosa, reflective of *Candida* esophagitis.

Despite clinical improvement, dietary adherence became increasingly challenging as the patient transitioned to a more typical diet. Consequently, treatment was transitioned to oral viscous budesonide (OVB) 1 mg daily, in conjunction with PPI therapy. Subsequent follow-up showed continued improvement in both esophageal eosinophilia and luminal caliber. Repeat endoscopy revealed complete histologic remission (0 eos/hpf) in the mid-esophagus and a step-off of mucosa at the site of the prior stricture (Figure 5). In addition, white specks were noted, which were consistent with *Candida* esophagitis and confirmed by a fungal culture. No further dilation was required (EoE HSS Grade 0/21, Stage 0/21). Candia esophagitis was treated with fluconazole.

## 3. Discussion and Conclusions

The concept of eosinophil-driven tissue remodeling in EoE parallels mechanisms observed in other chronic atopic diseases, including asthma. In both conditions, eosinophils promote T helper 2 (Th2)-associated cytokine production and release of profibrotic mediators, resulting in structural tissue changes such as airway remodeling and irreversible obstruction in asthma. In EoE, chronic mucosal injury and repeated cycles of repair initiate a remodeling cascade that ultimately leads to fibrosis.

Esophageal remodeling in EoE affects both the epithelial layer and the underlying lamina propria. Within the epithelium, eosinophil-derived products, such as major basic protein (MBP), promote basal zone hyperplasia via upregulation of fibroblast growth factor 9 (FGF-9) and increased epithelial proliferation. Transforming growth factor beta 1 (TGF-β1) further contributes by inducing epithelial–mesenchymal transition (EMT) and stimulating collagen I production, facilitating subepithelial fibrosis. Fibrosis of the lamina propria and smooth muscle hypertrophy are the primary drivers of esophageal rigidity and narrowing, largely mediated by IL-5, IL-13, and TGF-β1 [7].

Animal models have substantiated these mechanisms. In murine studies, intratracheal administration of anti–IL-13 antibodies protects against experimental EoE, whereas IL-13 instillation induces disease [8]. IL-13 overexpression alone is sufficient to provoke esophageal collagen deposition, tissue thickening, and edema, effects absent in mice lacking the IL-13 receptor [9]. Similarly, mice deficient in IL-5 or with GATA-1 promoter mutations (resulting in eosinophil deficiency) are protected against esophageal fibrosis following allergen exposure [10].

The long-term complications of EoE are largely attributable to tissue remodeling, which results in decreased esophageal compliance, increased stiffness, and stricture formation. These changes underlie common clinical manifestations such as dysphagia, food impaction, and feeding difficulties. Subepithelial fibrosis has been documented in children as early as infancy [11], with studies reporting its presence in 57%–89% of pediatric patients on diagnostic endoscopy [11, 12]. In addition, up to 88% of adults show subepithelial fibrosis at diagnosis [12]. In pediatric patients, symptoms including anorexia, early satiety, and dysphagia have been shown to correlate with histologic fibrosis, although fibrotic strictures are less commonly seen at younger ages. This supports the hypothesis that fibrotic sequelae develop gradually through ongoing eosinophilic inflammation.

Risk factors for fibrostenotic progression include older age, prolonged symptom duration prior to diagnosis, and higher symptom burden, particularly dysphagia and food impaction [2]. Data from a Swiss EoE registry identified symptom duration as a key predictor of stricture formation [12]. Moreover, inflammatory phenotypes tend to have a shorter symptom duration prior to diagnosis compared to mixed or fibrostenotic variants, suggesting a continuum of disease progression. Importantly, emerging evidence indicates that fibrostenotic changes are, to some extent, reversible with effective anti-inflammatory therapy, particularly when eosinophilic infiltration is reduced [13–15].

This case presents an unusual and early-onset form of stricturing EoE in a patient notable for both age and an atypical stricture location in the mid-esophagus (20 cm from the incisors), a site not typically associated with GER-related injury. To our knowledge, this is the youngest reported case of a mid-esophageal fibrostenotic stricture due to EoE. The absence of risk factors for caustic ingestion, congenital stricture, or other structural anomalies, as evidenced by a normal UGI series at 2 months of age, supports a diagnosis of primary EoE-related fibrostenosis. The presence of a stricture and significant esophageal eosinophilic inflammation despite high-dose PPI therapy further reinforces the diagnosis.

The patient responded well to serial balloon dilations, consistent with the existing literature on the safety and efficacy of dilation in pediatric EoE [1]. Mucosal inflammation improved with systemic corticosteroids, dietary elimination, OVB, and ongoing PPI therapy.

It is noteworthy that dietary elimination in this case was guided by atopy patch testing, reflecting clinical practice at the time (2017). However, current guidelines no longer recommend patch testing for dietary management due to low sensitivity and poor predictive value [16].

Although EoE is generally understood to evolve from an initial inflammatory phenotype to a later fibrostenotic phenotypeover time, with delayed diagnosis, male sex, and longer symptom duration as major risk factors, this case challenges the notion of a strictly linear disease trajectory. Instead, it raises the possibility that fibrostenosis may represent a distinct clinical phenotype, present early in the disease course in certain patients, or reflect an inherently more aggressive disease variant.

This case highlights the importance of maintaining a high index of suspicion for EoE in infants and toddlers presenting with feeding difficulties, particularly when symptoms persist or fail to respond to standard reflux therapy. Moreover, it suggests that EoE-related esophageal strictures can occur at younger ages than previously recognized.

## Data Availability

The data that support the findings of this study are available from the corresponding author upon reasonable request.
